# AuPd Bimetallic Nanocrystals Embedded in Magnetic Halloysite Nanotubes: Facile Synthesis and Catalytic Reduction of Nitroaromatic Compounds

**DOI:** 10.3390/nano7100333

**Published:** 2017-10-17

**Authors:** Lei Jia, Tao Zhou, Jun Xu, Fenghai Li, Zhouqing Xu, Beibei Zhang, Shengli Guo, Xiaoke Shen, Wensheng Zhang

**Affiliations:** 1 School of Chemistry and Chemical Engineering, Henan Polytechnic University, Jiaozuo 454000, China; jlxj@hpu.edu.cn (L.J.); zhoutaohpu@163.com (T.Z.); zhouqx@hpu.edu.cn (Z.X.); zbbhpu@163.com (B.Z.); guoshenglihpu@163.com (S.G.); shenxiaokehpu@163.com (X.S.); zhangwenshenghpu@163.com (W.Z.); 2 Collaborative Innovation Center of Coal Work Safety, Jiaozuo 454000, China; 3 School of Chemistry and Engineering, Heze University, Heze 274015, China; hzlfh@163.com

**Keywords:** halloysite nanotubes, bimetallic, 4-nitrophenol, magnetic, AuPd alloy

## Abstract

In this research, a facile and effective approach was developed for the preparation of well-designed AuPd alloyed catalysts supported on magnetic halloysite nanotubes (HNTs@Fe_3_O_4_@AuPd). The microstructure and the magnetic properties of HNTs@Fe_3_O_4_@AuPd were confirmed by transmission electron microscopy (TEM), high resolution TEM (HRTEM), energy-dispersive X-ray spectroscopy (EDS), and vibrating sample magnetometry (VSM) analyses. The catalysts, fabricated by a cheap, environmentally friendly, and simple surfactant-free formation process, exhibited high activities during the reduction of 4-nitrophenol and various other nitroaromatic compounds. Moreover, the catalytic activities of the HNTs@Fe_3_O_4_@AuPd nanocatalysts were tunable via adjusting the atomic ratio of AuPd during the synthesis. As compared with the monometallic nanocatalysts (HNTs@Fe_3_O_4_@Au and HNTs@Fe_3_O_4_@Pd), the bimetallic alloyed HNTs@Fe_3_O_4_@AuPd nanocatalysts exhibited excellent catalytic activities toward the reduction of 4-nitrophenol (4-NP) to 4-aminophenol. Furthermore, the as-obtained HNTs@Fe_3_O_4_@AuPd can be recycled several times, while retaining its functionality due to the stability and magnetic separation property.

## 1. Introduction

Noble metal nanoparticles (NPs), owing to their remarkable chemical and physical properties, have attracted significant attention in recent decades due to their potential use in electronics [1], optical devices [2], fuel cells [3], chemical sensors [4], catalysis [5,6,7] and biological materials [8,9,10]. Among these metals, gold nanoparticles (Au NPs) are frequently used for various catalytic reactions because Au catalyst is completely stable and active under mild condition [11,12]. For instance, Suvith et al. reported that Au NPs can be utilized for the catalytic degradation of methylene blue [13]. Toru Murayama et al. reported that nanoparticulate gold catalysts supported on niobium oxides (Nb_2_O_5_) are effective catalysts for CO oxidation [14]. Furthermore, bimetallic NPs have gained much attention due to the bi-functional properties generated from the monometallic component, with applications in optics [15], fuel cells [3], and catalysis [16,17,18]. Many studies demonstrated that the addition of palladium to gold or silver leads to more effective and high catalytic performance in reactions as compared to monometallic NPs. For example, an AuPd–MnO_x_/ZIF-8–rGO nanocatalyst prepared by a facile wet-chemical strategy showed highly efficient catalytic activity in the additive free dehydrogenation of formic acid [19]. In addition, Liu and co-workers reported that PdAg-N-doped-MOF-C can be used in catalytic transfer hydrogenation of nitro-compounds through the dramatically enhanced effect of Pd and Ag NPs in the reaction [20]. However, due to the high surface energy, free noble metal NPs aggregate easily, leading to a major reduction in catalytic activity and reusability. To solve this problem, many supporting materials, such as organic polymers materials [21], metal-organic framework [19], mesoporous silica [22], carbon materials [23], and so on [24,25], have been used to support and stabilize noble metal NPs.

Besides the above support materials, clay minerals are one of the more interesting nanostructured supports and are also regarded as a potential candidate for supports due to their excellent chemical and thermal stability. Among them, halloysite clay (HNT) is a natural and abundantly available nanoparticle formed by rolled kaolin sheets [26]. Very pure halloysite that possesses 90–98% of tubular structure (HNTs) is available in abundance only in France, Turkey, China, New Zealand, and USA [26,27]. Halloysite nanotubes (HNTs) are promising as a supporting material due to the inherent hollow tubular structure and charge differential between its inside and outside surfaces [28]. Due to its economical efficiency and biocompatibility [29,30,31,32], HNTs were recently studied for the development of innovative nanomaterials useful for catalytic [33,34] and biotechnological applications [35,36,37,38], with excellent geometrical and surface properties (large specific area, hollow tubular shape and tunable surface chemistry). In addition, when compared to other tubulose nanomaterials (such as boron nitride, metal oxide, and carbon nanotubes), HNTs are eco-compatible and non-toxic natural nanomaterials with remarkable and convenient applications in material science [26,27]. It is easy to imagine that in the near future HNTs could replace the much more expensive carbon nanotubes and in many cases, HNTs could be used in high technological applications where carbon nanotubes are just not suitable [26].

Moreover, researchers usually selected the reduction of 4-NP by sodium borohydride (NaBH_4_) as a model reaction to test the catalytic activity of various nanocatalysts [39,40]. The most likely reason is that 4-NP is one of the most hazardous contaminants, which is present in waste-waters due to the usage of pharmaceuticals, pesticides and dyes [41]. 4-NP can irreversibly damage the central nervous system, liver, and kidney of animals and humans. Therefore, the removal of 4-NP is important to protect the human being. Accordingly, several methods, such as photocatalytic degradation [42], microbial degradation [43], hazardous substances adsorption [44], and electrochemical degradation [45] have been developed to remove 4-NP. However, among the above techniques, the reduction of 4-NP to 4-aminophenol (4-AP) is the easiest way to remove 4-NP from the environment. The product 4-AP is a useful intermediate for applications of antipyretic and analgesic drugs [46]. So, due to the reasons of energy saving and safe operation, it is necessary to develop an effective, stable, easily available, and highly efficient catalyst for the reduction of 4-NP to 4-AP in aqueous solution under mild environments. Usually, noble metal formed nanocatalysts are difficult to recycle and reuse from reaction solution because of their small sizes and the looking for suitable catalyst carrier is very essential. HNTs@Fe_3_O_4_ is an ideal magnetic support [33], which is convenient to prepare and has more active sites for the anchoring of noble metal NPs. It can not only prevent the aggregation of noble metal NPs, but can also facilitate the recycle of nanocatalysts with the help of a magnetic field.

In this work, we reported a facile and convenient method to prepare the magnetic recoverable nanocatalysts HNTs@Fe_3_O_4_@AuPd. Firstly, the HNTs were coated by magnetite Fe_3_O_4_ particles through a one-pot solvothermal synthesis method. Secondly, the magnetic HNTs@Fe_3_O_4_ were further modified by the AuPd alloyed NPs or monometallic Au or Pd NPs. The catalytic activities of the above nanocatalysts toward the reduction reaction of 4-NP and its derivative in the presence of NaBH_4_ were investigated. The synergistic effect between Pd and Au endowed HNTs@Fe_3_O_4_@AuPd with superior activity when compared with monometallic HNTs@Fe_3_O_4_@Pd or HNTs@Fe_3_O_4_@Au catalysts. 

## 2. Results and Discussion

### 2.1. Structural and Morphology Characterization

**Figure 1:**
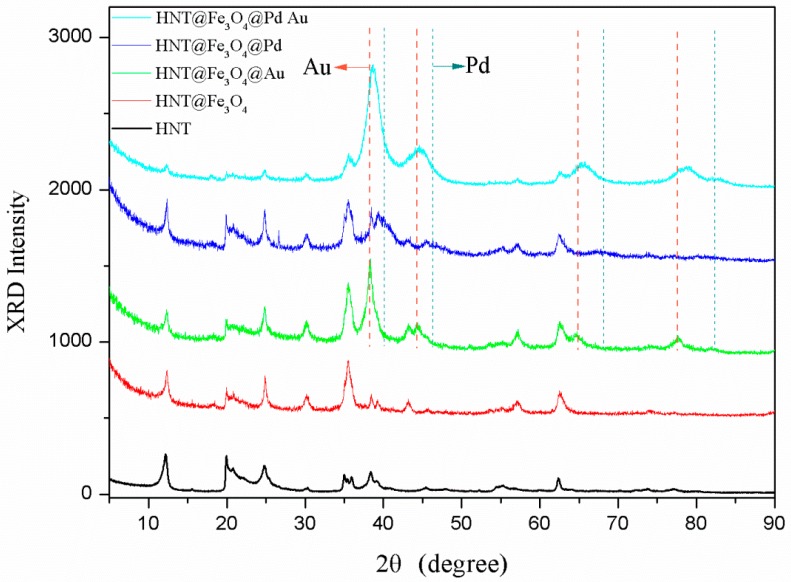
X-ray diffraction (XRD) patterns of as-synthesized nanocatalysts.

To investigate the formation of HNT@Fe_3_O_4_@AuPd nanocomposited catalysts, X-ray diffraction (XRD) analyses were conducted. Figure 1 represents the XRD patterns of HNT coated by Fe_3_O_4_, Au-Pd alloyed NPs, monometallic Au or Pd NPs, respectively. The exhibited characteristic diffraction peaks at 2θ = 12°, 20.3°, 24.9° originated from the HNTs for each sample, which demonstrate that the original inner structure of halloysite is not damaged during the whole preparation of HNT@Fe_3_O_4_@AuPd nanocatalysts. The observed XRD peaks at 2θ = 30.1°, 35.5°, 43.1°, 53.5°, 57.1° can be indexed to the planes of fcc Fe_3_O_4_ (JCPDS-19-0629) [47]. The monometallic HNT@Fe_3_O_4_@Au and HNT@Fe_3_O_4_@Pd composited nanoparticles exhibit wide diffraction peaks which well match to fcc structure of bulk Au (JCPDS-65-8601) [48] and bulk Pd (JCPDS-65-2867) [49]. The weak diffractions detected for Au and Pd from XRD patterns of the above bimetallic and monometallic nancatalysts indicate the formation of small NPs [50]. Furthermore, as compared to the (111) diffraction peak of Pd NPs in HNT@Fe_3_O_4_@Pd, the diffraction peaks of HNT@Fe_3_O_4_@AuPd shift to lower 2θ values towards the Au(111) peak, which is due to the increase of the lattice parameters and the formation of the crystalline Au-Pd alloy nanoparticles on HNT@Fe_3_O_4_ nanorods [19,51].

**Figure 2:**
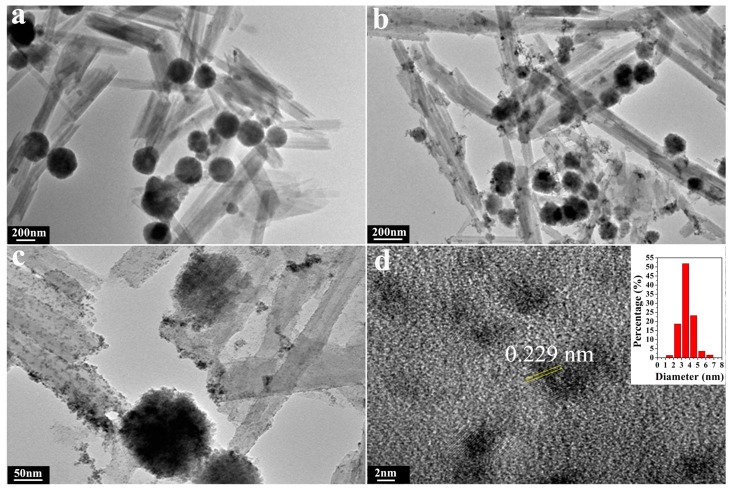
Transmission electron microscopy (TEM) images of HNTs@Fe_3_O_4_ (**a**), HNTs@Fe_3_O_4_@AuPd (**b**,**c**); high resolution TEM (HRTEM) image of AuPd bimetallic nanoparticles on the HNTs@Fe_3_O_4_ catalysts carrier (**d**); The inner image in (**d**) is the size distribution of AuPd bimetallic NPs.

**Figure 3:**
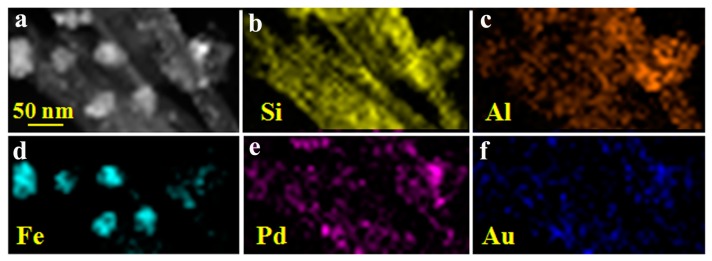
HAADF-STEM images of HNTs@Fe_3_O_4_@AuPd (**a**); energy-dispersive X-ray spectroscopy (EDX) mapping of Si element (**b**); Al element (**c**); Fe element (**d**); Pd element (**e**); and, Au element (**f**).

**Figure 4:**
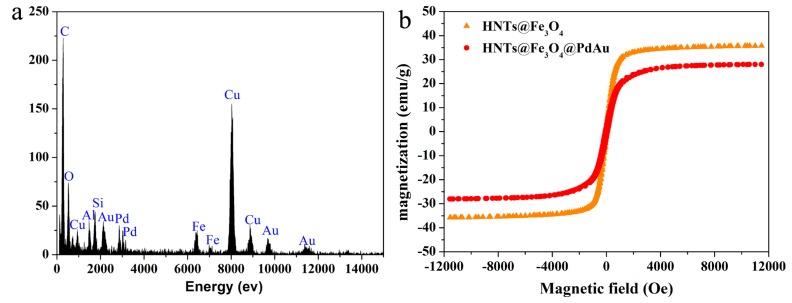
(**a**) EDX spectrum of HNTs@Fe_3_O_4_@AuPd nanocatalysts; (**b**) Room-temperature magnetization hysteresis loops of the as-prepared HNTs@Fe_3_O_4_ and HNTs@Fe_3_O_4_@AuPd.

The TEM images of HNTs@Fe_3_O_4_ and HNTs@Fe_3_O_4_@AuPd are shown in Figure 2. These images confirm that the nanocatalysts have been successfully fabricated and the HNTs still maintain their rod-like morphology during the preparation process. From Figure 2a, we can see the Fe_3_O_4_ nanoparticles (around 150 nm) are attached on the HNTs surface without obvious aggregation. AuPd bimetallic nanoparticles (diameter below 5 nm) have been successfully formed on the surface of HNTs@Fe_3_O_4_ by simply mixing the HAuCl_4_ and K_2_PdCl_4_ at room temperature. As shown in Figure 2b,c, there are many small nanoparticles adhered to the HNTs or Fe_3_O_4_ surface after the coreduction and there are no isolated AuPd catalysts outside the HNTs@Fe_3_O_4_. High-resolution TEM (HRTEM) (Figure 2d) and size distribution images indicate that the average size of AuPd bimetallic is 3.61 ± 0.28 nm. The chemical composition and elemental distribution of HNTs@Fe_3_O_4_@AuPd were investigated by EDS-mapping and the energy-dispersive X-ray spectroscopy (EDX). HAADF-STEM measurements (Figure 3) were carried out to investigate the elemental spatial distribution of Pd and Au. Here, it is necessary to mention that for the ultrafine AuPdNPs, while it is really difficult to get their HAADF-STEM results. Therefore, AuPd NPs with some aggregation were found by the HAADF-STEM test. The dark-field images indicate a abundant deposition of AuPd nanocatalysts on the magnetic support. Clearly, from Figure 3, Si, Al and Fe atoms are distributed on HNTs@Fe_3_O_4_, whilst Pd and Au atoms are distributed uniformly over the entire HNTs@Fe_3_O_4_@AuPd nanocatalysts, which provide a strong proof on the formation of alloy decorated nanocatalyst [51]. Furthermore, the typical HRTEM image (Figure 2d) of the AuPd NPs indicated that the interplanar spacing of the particle lattice is 0.229 nm, which approximated both the (111) lattice spacing of face centered cubic (fcc) Pd (0.223 nm) and Au (0.234 nm), and this may indicate that line defects are formed [52]. From the EDX image (Figure 4a), we can observe the existence of Si, Al, Fe, Au and Pd elements, which can also prove the successful deposition of Au and Pd on the HNTs@Fe_3_O_4_ catalysts carrier. According to the ICP-AES analyses, the content of the total metal supported on the HNTs@Fe_3_O_4_@AuPd nanocatalysts is 2.81 wt. % and the atomic ratio of Au:Pd is 0.402:0.598, which is consistent with the designed ratio. As shown in Figure 4b, the magnetic properties of HNTs@Fe_3_O_4_ and HNTs@Fe_3_O_4_@AuPd at room temperature demonstrate that the decreasing magnetization values can be assigned to the loading of non-magnetic AuPd nanoparticles. However, the current magnetism is still strong enough for the recycling and reusing of the catalysts during the whole catalytic experiments.

### 2.2. Catalytic Activities of Bimetallic or Monometallic Au/Pd Modified Nanocatalysts in the Reduction of 4-NP

**Figure 5:**
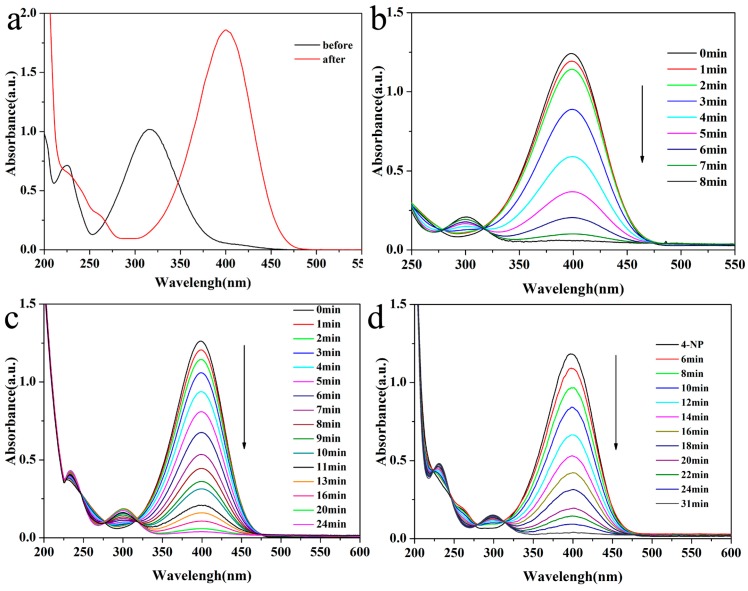
(**a**) UV-vis spectra of 4-NP before and after adding NaBH_4_ solution without catalysts; time-dependent UV-vis absorption spectra of reduction of 4-NP by NaBH_4_ in presence of HNT@Fe_3_O_4_@Au_40_Pd_60_ (**b**); HNT@Fe_3_O_4_@Pd (**c**) and HNT@Fe_3_O_4_@Au (**d**).

The catalytic activities of as-prepared bimetallic and monometallic nanocatalysts were tested by the catalysts for the reduction of 4-NP with an excess of NaBH_4_, as depicted in Figure 5. The conversion from 4-NP to 4-AP can be easily monitored by the UV–vis spectroscopy. Generally, the UV–Visible spectra of 4-NP solutions have a distinct spectral profile with a maximum absorption peak at 317 nm in neutral or acidic situation, which is presented in Figure 5a. After addition of NaBH_4_, the absorption band shifts to 400 nm immediately, accompanied with the color change from light yellow to bright yellow. This peak shift is due to the formation of 4-nitrophenolate ion as the alkalinity of the solution increased [39]. Without the addition of the catalysts, the maximum absorption peak at 400 nm can not be altered, indicating that there is no reduction reaction, which is caused by the high kinetic barrier between 4-nitrophenolate ion and BH_4_^−^ ion. 

After the addition of a small amount of HNT@Fe_3_O_4_@Au_40_Pd_60_ into the 4-NP and NaBH_4_ mixed solution, the color of the solution changed from bright yellow to colorless quickly, signalling the completion of the reaction. Time-dependent absorbance spectra of this reaction shows the intensity of the absorption spectra at 400 nm gradually decrease along with a concomitant increase of the 300 nm peak of 4-aminophenol, revealing the formation of 4-AP [51]. After 8 min, the peak at 400 nm is no longer observed, indicating that the reduction reaction is completed (Figure 5b). We proposed that BH_4_^−^ would adsorb onto the surface of HNT@Fe_3_O_4_@Au_40_Pd_60_ and divert activated hydrogen to the AuPd active centre to form a metal hydride complex [53]. Meanwhile, 4-NP can also simultaneously stick to the surfaces of HNT@Fe_3_O_4_@Au_40_Pd_60_ via chemical adsorption [54]. In the above factors, the transformation of adsorbed hydrogen species to 4-NP can reduce the nitro groups to amino groups, which is strongly supported by previous results [55,56].

**Figure 6:**
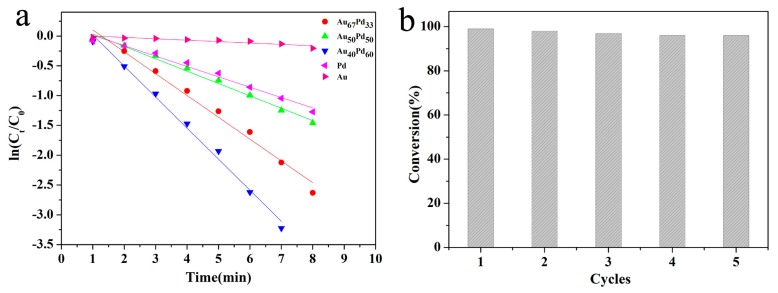
(**a**) The relationships between ln(C_t_/C_0_) and reaction time t (d) for the reduction of 4-NP over different catalysts; (**b**) the reusability of the Au_40_Pd_60_@HNT@Fe_3_O_4_ nanocatalysts for the catalytic reduction of 4-NP.

For a comprehensive elucidation of the catalytic property of the HNT@Fe_3_O_4_@Au_40_Pd_60_ and the synergetic effects of Au and Pd catalytic architecture in the reduction of 4-NP, we investigated the catalytic behaviour of monometallic HNT@Fe_3_O_4_@Au, HNT@Fe_3_O_4_@Pd, and bimetallic HNT@Fe_3_O_4_@Au_40_Pd_60_ with different molar compositions of Au versus Pd under the identical conditions (the amount of each catalyst is controlled at 5 μg). As shown in the time-dependent UV-vis absorption spectra of Figure 5c,d, the time for HNT@Fe_3_O_4_@Pd to finish the reduction is slight shorter than that for HNT@Fe_3_O_4_@Au, while the time of the two catalysts used are obvious longer than that for HNT@Fe_3_O_4_@Au_40_Pd_60_. These results indicate that the catalytic activity of magnetic HNT-based AuPd alloyed bimetallic nanocatalysts is evidently enhanced as compared to the Au or Pd monometallic nanocatalysts. As a matter of fact, the catalytic activities of the HNT@Fe_3_O_4_@AuPd nanocomposite are tunable by altering the Au versus Pd atomic ratios. The catalytic reactions own the highest reaction rate when the atomic ratio of the alloyed catalysts reaches Au_40_Pd_60_, which can be understood in Figure 6a.

To further evaluate the catalytic activities of the above different nanocatalysts, a pseudo-first order kinetics equation was applied, which is due to the amount of BH_4_^−^ in the catalytic system is more excessive than that of 4-NP and the reaction rate is not affected by the concentration of BH_4_^−^. The experiment was carried out under the same conditions and the rate constant (k) is calculated as: k_ap_t = ln(C_t_/C_0_), where C_0_ is the initial absorbance of the reagents at the maximum absorption wavelength, C_t_ is the absorbance of reagents at maximum absorption wavelength under different time t and K_ap_ is the apparent rate constant. Figure 6a shows the approximately linear relationships of ln(A_t_/A_0_) vs. reaction time in the reaction catalyzed by the as synthesized nanocatalysts. It can be clearly seen that the reactions follow the pseudo-first-order reaction kinetics. Hence, the apparent rate constants obtained from the slopes of the linearly plots are 0.520, 0.389, 0.216, 0.023, and 0.174 min^−1^ for the HNT@Fe_3_O_4_@Au_40_Pd_60_, HNT@Fe_3_O_4_@Au_67_Pd_33_, HNT@Fe_3_O_4_@Au_50_Pd_50_, HNT@Fe_3_O_4_@Au and HNT@Fe_3_O_4_@Pd, respectively. It is obvious that all the HNT@Fe_3_O_4_@Au_40_Pd_60_ nanocatalysts have the highest catalytic ability and its reaction rate is about 22.61 and 2.98 times faster than that of the monometallic Au or Pd modified nanocatalysts, which is accordance with the results obtained from the time-dependent UV-vis absorption spectra.

Furthermore, the scope of the catalytic activity of HNT@Fe_3_O_4_@Au_40_Pd_60_ in the reduction for other nitroaniline derivates was also investigated. All the catalytic conditions of these analogues are same as that of 4-NP and the reaction progress of the nitroaniline was monitored by UV-vis absorption spectrometry. The data of reaction time and the conversion were calculated and are listed in Table 1, revealing our catalysts have excellent catalytic activities with perfect yield toward nitrophenol and nitroaniline derivatives regardless of the types and position of the substituents. Four kinds of nitroaniline can be reduced within 8 min with a conversion more than 99%. Interestingly, when the HNT@Fe_3_O_4_@Au_40_Pd_60_ was used to reduce the nitrotoluene analog substrate, it displayed a lower activity than those of nitroaniline and nitrophenol, which indicated that the reaction processes of nitrotoluene derivatives are more complicated than those of nitroaniline and nitrophenol derivatives [57].

### 2.3. Reusability of the HNT@Fe_3_O_4_@Au_40_Pd_60_

It is well known that the reusability is a main advantage of using a heterogeneous catalyst rather than a homogeneous catalyst. As the Fe_3_O_4_ NPs have remarkable magnetic properties [58], the catalysts were recovered by simple magnetic separation with the help of a magnetic field after completion of the reduction reaction and then washed with water for reusing in the next cycle. The reusability of the HNT@Fe_3_O_4_@Au_40_Pd_60_ were studied for the reduction of 4-nitrophenol with NaBH_4_. As shown in Figure 6b, even after five successive recycle, HNT@Fe_3_O_4_@Au_40_Pd_60_ still displayed excellent conversion efficiency without significant loss of its activity. Besides the catalytic activity and reusability, the stability of the catalysts is also a very important issue for industrial applications. To examine the stability of the nanocatalysts, the reduction reaction was repeated using composited catalysts for more than three months. The conversion of 4-NP was similar after the catalysts are prepared three months later, suggesting that the catalysts were stable and can be used within three months without an obvious loss of activity [59].

## 3. Materials and Methods 

### 3.1. Materials

Halloysite was purchased from China-Kaolinite Company (Suzhou, China). Ferric chloride hexahydrate (FeCl_3_·6H_2_O), sodium acetate (NaAc), polyethylene glycol (PEI), ethylene glycol (EG), potassium tetrachloropalladate (K_2_PdCl_4_)m and hydrogen tetrachloroaurate (HAuCl_4_) were obtained from Sinopharm Chemistry Reagent Co., Ltd. (Beijing, China). 4-nitrophenol, p-nitroaniline, m-nitroaniline, o-Nitroaniline (o-NA), 2,4-Nitroaniline and sodium hydroxide (NaBH_4_) were bought from Sigma Aldrich. All of the chemicals were of chemical reagent grade and used without any further purification. Ultrapure water was prepared by using NANO Pure Infinity System (Barnstead/Thermolyne Corp, Dubuque, IA, USA).

### 3.2. Preparation of HNT@Fe_3_O_4_

HNT coated by Fe_3_O_4_ were synthesized through a solvothermal reaction according to a previously described method with some modification [60]. Briefly, 1 g of FeCl_3_·6H_2_O was added to 30 mL of ethylene glycol. A clear yellow solution was obtained after sonication for 3 min. Then, 2.7 g of sodium acetate (NaAc) and 0.75 g PEI were added to the above solution and stirred for another 0.5 h. Subsequently, 0.3 g HNT was added. When the mixed solution was ultrasonically dispersed for 3 h to form a homogeneous dispersion, the mixture solution was transferred to a Teflon-lined stainless-steel autoclave (50 mL capacity). The autoclave was sealed and maintained at 200 °C. After reaction for 8 h, the autoclave was naturally cooled to ambient temperature. The obtained HNT@Fe_3_O_4_ black magnetic particles were collected by an external magnetic field and washed four times with ethanol and deionized water in sequence. The product was then dried in vacuum at 60 for 12 h.

### 3.3. Preparation of HNTs@Fe_3_O_4_@AuPd, HNTs@Fe_3_O_4_@Au and HNTs@Fe_3_O_4_@Pd

In a typical synthesis of HNTs@Fe_3_O_4_@AuPd, 5.0 mL aqueous solution containing HNT@Fe_3_O_4_ (50 mg), HAuCl_4_ (0.5 mL, 10 mM), and K_2_PdCl_4_ (0.5 mL 10 mM) was kept in a glass bottle under stirring for 3 h. Then, the fresh 1.0 mL aqueous solution NaBH_4_ (28 mg, 0.7 mmol) was added and the resulting solution was stirred for another 0.5 h at ambient temperature. Then, the reaction mixture was centrifuged and washed with pure water to remove the remaining reagents. For comparison, other catalysts were also prepared using the same method: (1) HNT@Fe_3_O_4_ (50 mg) and HAuCl_4_ (0.5 mL, 10 mM) for HNTs@Fe_3_O_4_@Au, (2) HNT@Fe_3_O_4_ (50 mg) and K_2_PdCl_4_ (0.75 mL, 10 mM) for HNTs@Fe_3_O_4_@Pd and (3) HNT@Fe_3_O_4_ (50 mg), 0.5 mL 10 mM HAuCl_4_ and v mL 10 mM K_2_PdCl_4_ (v = 0.25, 0.75) for HNTs@Fe_3_O_4_@Au_x_Pd_y_.

### 3.4. Catalytic Reduction of Nitrobenzene

The reduction of 4-NP with NaBH_4_ was chosen to examine the catalytic activity and reusability of the HNTs@Fe_3_O_4_@Au_40_Pd_60_ catalysts. In a typical procedure, 1 mL of 4-NP (0.1 mM) and 1.0 mL of fresh NaBH_4_ (10 mM) was taken in a quartz cuvette, followed by addition of 20 μL of catalysts (0.25 mg/mL) to the mixture. The reaction solution was immediately monitored using UV–vis spectrophotometer at 1 min interval. The color of the solution changed gradually from yellow to colorless. The catalytic reduction of other nitroaniline were conducted under the same condition of 4-NP. Following the similar procedures, HNT@Fe_3_O_4_@Au, HNT@Fe_3_O_4_@Pd, and HNT@Fe_3_O_4_@Au_x_Pd_y_ (each of 5 μg) were also used as catalysts for the reduction of 4-NP. 

### 3.5. Characterization

Transmission electron microscopy (TEM), high resolution transmission electron microscopy (HRTEM), and the energy dispersive spectra (EDS) were determined by a Tecnai-G2-F30 at acceleration voltages of 200 kV. X-ray diffraction (XRD) measurements were carried out on a X’pert PRO X-ray power diffractometer using Cu Ka radiation of 1.5406 A (40 kV, 30 mA). Magnetization measurements were performed on a vibrating sample magnetometry (VSM, LAKESHORE-7304, Westerville, OH, USA) at room temperature. The UV measurement was finished on a Shimadzu UV-240 spectrophotometer. Au, Pd contents of the samples were determined by inductively coupled plasma-atomic emission spectroscopy (ICP-AES) using an IRIS Advantage ER/S spectrophotometer.

## 4. Conclusions

In conclusion, we fabricated magnetically separable and well dispersed bimetallic Au_40_Pd_60_ alloyed nanoparticles on HNT@Fe_3_O_4_ by a facile method. The HNT@Fe_3_O_4_@Au_40_Pd_60_ nanocatalysts were explored for the reduction of organic pollutants in an aqueous medium. When compared with the monometallic HNT@Fe_3_O_4_@Au and HNT@Fe_3_O_4_@Pd nanocatalysts prepared under the same conditions, the AuPd alloyed HNT@Fe_3_O_4_@Au_40_Pd_60_ nanocatalysts exhibited the much better catalytic performance, which may be ascribed to the synergistic effects of Pd and Au. Furthermore, the HNT@Fe_3_O_4_@Au_40_Pd_60_ catalysts can be easily separated from the reaction system with the help of an external magnet and reused several cycles without significant loss of the activity. The as-obtained hybrids may become ideal recyclable catalysts for the reduction of aromatic nitro compounds owing to their stability and efficient magnetism.

## Figures and Tables

**Table 1:** Reduction of various nitrobenzenes using HNT@Fe_3_O_4_@Au_40_Pd_60_ catalysts.

Entry	Compound	Time/min	Conversion/%
**1**	p-Nitroaniline	8	99
**2**	m-Nitroaniline	6	99
**3**	o-Nitroaniline	5	99
**4**	2,4-Nitroaniline	7	99
**5**	m-Nitrotoluene	68	81
**6**	o-Nitrotoluene	76	75
**7**	2,4-Dinitrotoluene	82	79
